# Tracking Research Momentum and Scholarly Impact: A Bibliometric Analysis of Magnetotherapy

**DOI:** 10.7759/cureus.69243

**Published:** 2024-09-12

**Authors:** Vinoj M N, Resmi S, Priyadarsini G, Seenamol K Stephen, I John Berlin, Jobin Jose

**Affiliations:** 1 Department of Physics, St. Peter's College, Kolenchery, IND; 2 Department of Physics, Sree Narayana College for Women, Kollam, IND; 3 Department of Physics, Government Polytechnic College Kaduthuruthy, Kaduthuruthy, IND; 4 Department of Physics, Mar Thoma College, Tiruvalla, IND; 5 Department of Library Science, Marian College Kuttikkanam (Autonomous), Kuttikkanam, IND

**Keywords:** bibliometric analysis, biblioshiny, citespace, magnetic field therapy, magnetic therapy, magnetotherapy

## Abstract

This bibliometric study investigates the research landscape of magnetotherapy, also known as magnetic field therapy, which utilizes magnetic fields to promote healing, reduce pain, and improve overall health and well-being. The study uses bibliographic data from Scopus and employs Biblioshiny and CiteSpace for analysis. Key aspects examined include annual scientific production, most relevant authors, co-citation of cited authors, most relevant sources, countries' scientific production, and collaborations. Additional analyses feature a historiograph, trend topics, thematic maps, and keywords with the strongest citation bursts. Findings highlight significant trends in research activity, identify influential contributors, and map out collaborative networks in the field. The study reveals a noticeable absence of long-term studies and standardized treatment protocols in magnetotherapy research. Identified research gaps indicate the need for interdisciplinary studies and a deeper mechanistic understanding of magnetotherapy. Practical implications emphasize the potential for enhanced clinical applications, healthcare policy integration, and medical device innovations. The study underscores the importance of educational and training programs for healthcare professionals. Addressing these gaps and leveraging practical implications can significantly advance the field of magnetotherapy.

## Introduction and background

Magnetotherapy, also known as magnetic field therapy, involves using magnetic fields to improve health and well-being [1,2]. It is used to stimulate physical responses that can alleviate pain and treat various diseases [2,3]. Despite its widespread use, there is still limited and often disputed scientific evidence regarding its effectiveness. There are multiple methods of magnetotherapy that utilize different types of magnetic fields. Static magnetic field therapy involves using magnets applied to the skin, such as magnetic bracelets, magnetized mattress pads, and magnetic patches [4]. These static fields are believed to interact with the body's bioelectromagnetic fields and can have cellular and physiological effects [5]. Electromagnetic therapy uses charged magnets and typically delivers electric pulses [6]. Devices such as pulsed electromagnetic field (PEMF) therapy machines generate dynamic magnetic fields capable of penetrating deeper into tissues, increasing blood flow, reducing inflammation, and enhancing cellular-level repair [6]. Finally, magnetic therapy with acupuncture applies magnets to specific acupuncture points on the skin, combining acupuncture concepts with magnetic fields to rebalance the body's energy flow (Qi) and stimulate the healing process [7].

Magnetotherapy is used to treat various disorders, such as relieving arthritis pain, healing wounds, reducing sleeplessness, alleviating headaches, and decreasing fibromyalgia pain [8]. In cases of arthritis, it helps in reducing pain and inflammation in the joints. With wound healing, it promotes faster recovery and tissue regeneration. For sleeplessness, it improves the quality of sleep, possibly by affecting the body's biorhythm [9]. It is also used to lessen the frequency and intensity of migraines and tension headaches and to alleviate chronic pain and fatigue associated with fibromyalgia [10]. Recent advancements in magnetotherapy are focused on enhancing the precision, effectiveness, and accessibility of treatments [11]. PEMF therapy has seen improvements, with devices now being developed with adjustable frequencies and intensities for personalized treatment plans [12]. Studies indicate that it might help in pain management, bone healing, and post-operative surgery recovery [13]. Wearable magnetic devices, such as magnetic insoles, wristbands, and back supports, have been created, though trial results are mixed regarding their effectiveness [14]. Its integration into the digital health field has led to the development of smart magnetotherapy devices connected to a mobile app, which could monitor and adapt treatments. Ongoing trials are evaluating their effectiveness in managing chronic conditions [11].

The effectiveness of magnetotherapy in clinical settings is currently subject to debate. While some individual reports and studies suggest possible benefits, there is limited scientific evidence to support its widespread use [15]. When it comes to managing pain, results from clinical trials have been inconsistent, with some showing a significant reduction in pain and others indicating minimal effects [16]. Proposed mechanisms for how magnetotherapy works include increased blood flow, reduced inflammation, and the release of endorphins. PEMF therapy has been found to aid in the healing of fractures and in increasing bone density by enhancing the activity of osteoblasts and promoting calcium uptake [17]. Some studies also suggest that magnetic fields may have the potential to modulate inflammatory responses.

Magnetotherapy has multiple benefits, including being non-invasive with minimal side effects and providing significant pain relief, which can be a valuable addition to traditional treatments [18]. However, there are significant challenges associated with magnetotherapy, including the lack of thorough scientific evidence and standardized protocols, the potential for bias from the placebo effect in reported outcomes, and inadequate regulation and variable quality of magnetic therapy devices [19]. While magnetotherapy shows promise, more comprehensive scientific research is necessary to validate its mechanisms and effectiveness [20]. Technological advancements and digital health may pave the way for more efficient and tailored magnetic treatment solutions in the near future.

Bibliometric analysis is a crucial method for evaluating the extensive body of research literature on magnetotherapy. It provides insights into the field's development and trends [21,22]. Tools such as Biblioshiny and CiteSpace can be used to comprehensively analyze publication data to identify leading studies, prolific authors, and key research themes [23,24]. Biblioshiny is a user-friendly, web-based application designed for in-depth analysis and visualization of bibliometric data, making it easier to detect trends and patterns in the literature [25]. CiteSpace, on the other hand, is a Java-based application that excels in visualizing citation networks and discovering the intellectual structure and dynamic evolution of research fields [26,27]. Together, these tools offer a comprehensive framework for understanding the scholarly landscape of magnet therapy.

Bibliometric analysis using Biblioshiny and CiteSpace can provide important insights into the development path of magnetotherapy, potential collaboration networks, and emerging research areas in the field. This approach can help identify significant contributions in the literature, leading researchers and institutions, and track the dissemination of important findings. By analyzing citation patterns and co-citation networks, we can trace key foundational studies and theoretical frameworks that have influenced magnetotherapy research. Such a systematic approach will enhance our understanding of the field and contribute to shaping future research directions and clinical practices, ultimately advancing magnetotherapy as a treatment method.

## Review

Materials and methods

**Figure 1:**
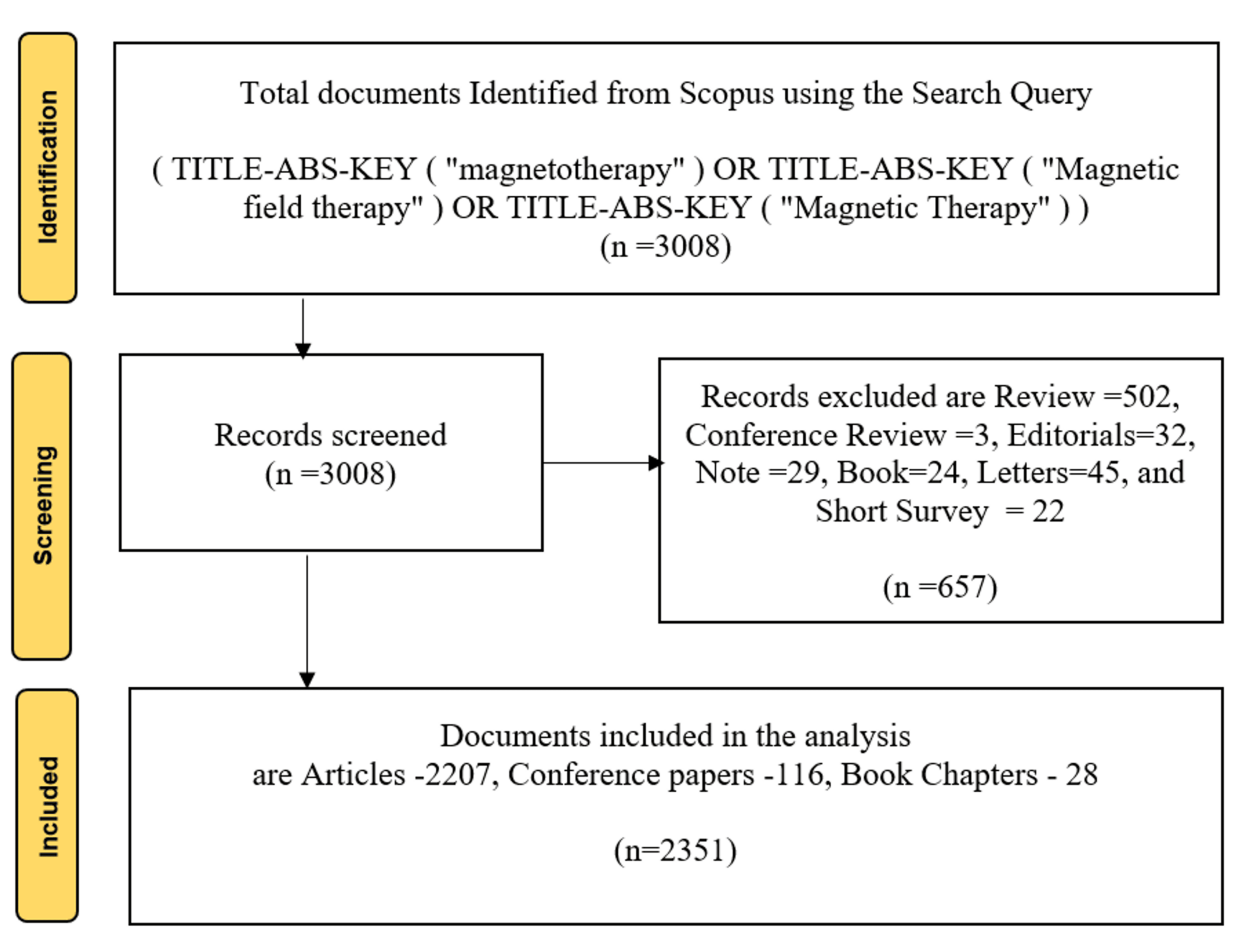
PRISMA flow chart The flowchart illustrates the systematic review process, starting with 3008 documents identified from Scopus, screening out 657 records (including reviews, editorials, and short surveys), and ultimately including 2351 documents (articles, conference papers, and book chapters) in the analysis. PRISMA: Preferred Reporting Items for Systematic Reviews and Meta-Analyses

The primary bibliographic data source for this study is Scopus because it includes a wider range of high-quality journals compared to other databases [28]. We retrieved publications using the keywords "magnetotherapy," "Magnetic field therapy," or "Magnetic Therapy" with no language restrictions and considered only journal articles, conference papers, and book chapters. A total of 2351 documents were collected from 1139 different sources spanning from 1945 to 2024. Figure 1 illustrates the Preferred Reporting Items for Systematic Reviews and Meta-Analyses (PRISMA) approach to selecting papers for bibliometric analysis, which involves a three-phase procedure. In the first phase, we identified and extracted the data from the databases. In the second phase, we excluded reviews, editorials, books, letters, notes, and short surveys and only included articles, conference papers, and book chapters. The findings were stored as "CSV" and RIS files, and we performed bibliometric analysis on the data using CiteSpace version 6.2. R3 (Advanced) and Bibloshiny software.

Main Information of the Investigation

Table 1 provides the key findings from the bibliometric analysis of magnetotherapy. The analysis covers the period from 1945 to 2024 and includes 1139 sources such as journals, books, and conference proceedings. During this time, 2351 documents were published, demonstrating significant research activity with an annual growth rate of 5.23%. The average age of the documents is 12.1 years, indicating a mix of historical and current research. On average, each document receives 15.99 citations, illustrating their impact, with a total of 61,160 references cited. An analysis of the document content reveals 13,689 keywords plus (ID) and 4,582 author's keywords (DE), indicating a wide range of research topics. The authorship involves 8878 contributors, with 156 single-authored works, reflecting a collaborative research environment where the average number of co-authors per document is 4.97, and 11.82% of documents involve international collaborations. Document types include 2,207 articles, 28 book chapters, and 116 conference papers, indicating that peer-reviewed journals are the primary medium of dissemination, complemented by academic books and conference presentations.

**Table 1:** Main information of the investigation

Description	Results
Main Information About Data	
Timespan	1945:2024
Sources (Journals, Books, etc.)	1139
Documents	2351
Annual Growth Rate %	5.23
Document Average Age	12.1
Average citations per doc	15.99
References	61160
Document Contents	
Keywords Plus (ID)	13689
Author's Keywords (DE)	4582
Authors	
Authors	8878
Authors of single-authored docs	156
Author's Collaboration	
Single-authored docs	174
Co-Authors per Doc	4.97
International co-authorships %	11.82
Document Types	
Article	2207
Book chapter	28
Conference paper	116

Annual Scientific Productions

**Figure 2:**
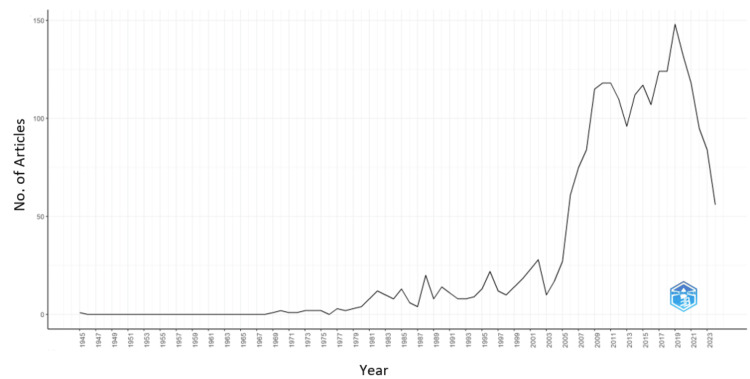
Annual scientific production from 1945 to 2024 The figure shows the annual scientific production from 1945 to 2024, highlighting a significant increase in the number of articles published in recent years.

The field of magnetotherapy has shown significant fluctuations and growth in annual scientific production over the years, as illustrated in Figure 2. The earliest documented publication in 1945 was followed by a long period of inactivity, with no publications recorded until 1969. From 1969 onward, there was a gradual increase in scientific output, with occasional years of no publications. The 1970s and early 1980s marked a slow but steady rise, with the number of articles per year remaining in the single digits or low teens. A notable surge in research activity began in the late 1980s, particularly from 1988 onwards, with a sharp increase in 20 publications. The trend continued to accelerate throughout the 1990s and into the 2000s, with a significant jump in annual publications starting in 2005, which saw 27 articles. This upward trajectory continued, peaking in 2019 with the highest number of publications at 148 articles. This peak reflects a heightened interest and a substantial increase in research activities related to magnetotherapy. The trend maintained relatively high levels of output from 2016 to 2021, with over 100 articles annually. There was a slight dip to 95 articles in 2022, followed by 84 in 2023. Up to June 2024, there has already been a substantial count of 56 publications, indicating that the final count for the year is likely to be significant, continuing the robust research interest in this field.

Most Relevant Authors

Table 2 highlights the most relevant authors in magnetotherapy research based on their publication counts. Leading the field is Zhang Y with 22 articles, followed by Sieroaf A with 20 articles, and Li Y with 19 articles. Bolotova NV and Pasek J each have 18 articles, while Daskalakis ZJ, Wang J, and Wang Y have each contributed 17 articles. Additionally, Zhang X and Zhou J have each published 16 articles. These authors are key contributors to the field, with their significant publication records indicating their influence and active involvement in advancing magnetotherapy research.

**Table 2:** Most relevant authors The table lists the most relevant authors in the field, ranked by the number of articles they have published

Authors	Articles
Zhang Y	22
Sieroåƒ A	20
Li Y	19
Bolotova NV	18
Pasek J	18
Daskalakis ZJ	17
Wang J	17
Wang Y	17
Zhang X	16
Zhou J	16

Network Visualization of Co-citation of Cited Authors

**Figure 3:**
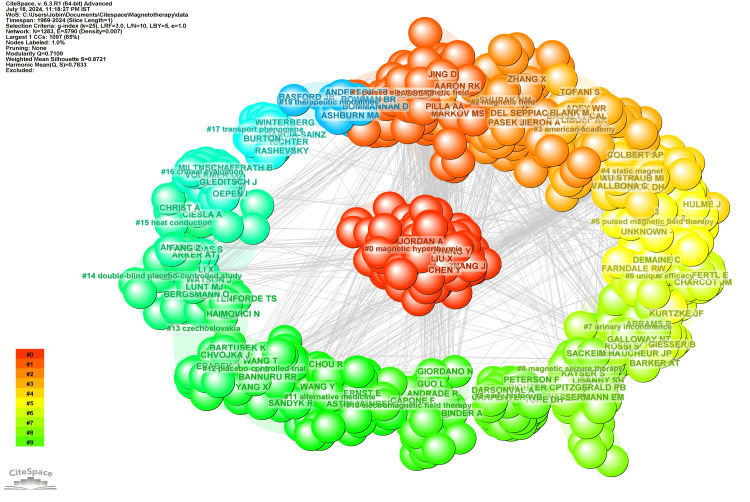
Network visualization of co-citation of cited authors The figure displays a network visualization of co-citation among cited authors, where the size of the nodes represents the frequency of citations, and the color gradient from red to green indicates the strength of co-citation relationships, with clusters highlighting closely related groups of authors within the research field.

Figure 3 presents a detailed network visualization of co-citation among cited authors in the realm of research. This visualization highlights how frequently authors are cited together in other works, uncovering the underlying collaboration patterns and intellectual structure within this research area [29].

The network consists of 22 distinct clusters, each representing a unique subfield within magnetotherapy, showcasing the diversity and interconnectedness of research topics in this domain. The largest cluster, with 151 members, is labeled Magnetic Hyperthermia and has a silhouette value of 0.749. This cluster is centered around the use of magnetic fields to generate heat in targeted areas, a technique primarily used in cancer therapy. Key authors include Zhang Y, Jordan A, and Zhang J. Cluster #1, with 142 members and a silhouette value of 0.72, focuses on pulsed electromagnetic fields. This research area investigates the therapeutic potential of pulsed electromagnetic fields in treating various medical conditions. Key contributors are Markov MS, Bassett CA, and Pilla AA. The third-largest cluster, Magnetic Field, has 106 members and a silhouette value of 0.825. This cluster explores the effects of magnetic fields, particularly in models of chronic neuropathic pain. The work of Sieron A, Shupak NM, and Zhang X is prominent in this cluster. Cluster #3, labeled American Academy, consists of 76 members and has a silhouette value of 0.899. This cluster examines the historical and contemporary techniques in pulsed electromagnetic field therapy. Key authors like Liboff AR and Bassett CAL have contributed significantly to this area, particularly in understanding the evolution of these techniques.

With 75 members, Cluster #4 is focused on static magnet therapy and has a silhouette value of 0.861. This cluster explores the use of static magnets in therapeutic contexts, particularly in managing inflammatory conditions. Weintraub MI and Trock DH are key contributors to this cluster, advancing the understanding of how static magnetic fields can influence health outcomes. Cluster #5, with 60 members and a silhouette value of 0.876, is labeled Pulsed Magnetic Field Therapy. This cluster focuses on the application of pulsed magnetic fields in treating various medical conditions, including bone fractures like tibial non-union. The 7th largest cluster, Unique Efficacy, has 60 members and a high silhouette value of 0.974. This cluster investigates the efficacy of extremely low-intensity magnetic fields, particularly in treating neurological conditions like Parkinson's disease. Cluster #7, labeled Urinary Incontinence, comprises 58 members and has a silhouette value of 0.917. This cluster explores the use of magnetic fields in treating urinary incontinence and other related conditions. Authors like Barker AT and Lefaucheur JP have made significant contributions to this cluster, advancing the understanding of how magnetic fields can be used in neuromodulation.

The 9th largest cluster, Magnetic Seizure Therapy, has 47 members and a silhouette value of 0.929. This cluster focuses on the use of magnetic fields in seizure therapy, particularly for treating major depressive disorder. Key contributors include Lisanby SH and Fitzgerald PB, who have explored the therapeutic potential of magnetic fields in mental health. Cluster #9, labeled Early History, consists of 41 members and has a silhouette value of 1. This cluster examines the historical development of electrodermal research, particularly in the context of electromagnetic fields. Authors like Tuke DH and Carpenter WB have contributed to this cluster, providing valuable insights into electromagnetic field research's origins and evolution. The 11th largest cluster, Electromagnetic Field Therapy, has 41 members and a silhouette value of 0.93. This cluster investigates the therapeutic use of electromagnetic fields, particularly in managing pain and other symptoms following dental surgery. Authors like Binder A and Guo L have made significant contributions to this cluster, advancing the understanding of how electromagnetic fields can be used in pain management. Cluster #11, labeled Alternative Medicine, consists of 35 members and has a silhouette value of 0.95. This cluster explores the use of alternative therapies, including electromagnetic fields, in treating musculoskeletal pain. Key contributors include Eisenberg DM and Wang Y, who have explored the integration of alternative medicine with conventional treatments. Cluster #12, with 31 members and a silhouette value of 0.94, is labeled Placebo-Controlled Trial. This cluster focuses on the rigorous evaluation of electromagnetic field therapies through placebo-controlled studies. Authors like Sandyk R and Li S are prominent in this cluster, contributing to the robust assessment of electromagnetic therapies.

The 14th largest cluster, Czechoslovakia, has 30 members and a silhouette value of 1. This cluster examines the development and application of magnetotherapy in Czechoslovakia. Jerabek's 1994 review of pulsed magnetotherapy in Czechoslovakia is a key reference, reflecting the country's pioneering role in this field [30]. Contributors like Tenforde TS and Haimovici N have played significant roles in this cluster, highlighting the historical and regional developments in magnetotherapy. Cluster #14, labeled Double-Blind Placebo-Controlled Study, has 29 members and a silhouette value of 0.95. This cluster focuses on double-blind, placebo-controlled studies of electromagnetic field therapies, particularly in treating conditions like insomnia. Authors like Barker AT and Watson J have made important contributions to this cluster, which emphasizes the importance of rigorous clinical trials in validating the effectiveness of electromagnetic therapies. Cluster #15, with 25 members and a silhouette value of 0.974, is labeled Heat Conduction. This cluster explores the interaction between magnetic fields and heat conduction, particularly in the context of blood flow models. Contributors like Li X and Ciesla A have made significant contributions to understanding how magnetic fields influence heat conduction in biological systems.

The 17th largest cluster, Critical Evaluation, consists of 24 members and has a silhouette value of 1. This cluster critically evaluates unconventional diagnostic and therapeutic methods, including those involving electromagnetic fields, in dentistry. Authors like Miltner W and Volkmer D have contributed to this cluster, which emphasizes the need for rigorous scrutiny of non-traditional therapeutic approaches. Cluster #17, labeled Transport Phenomena, has 18 members and a silhouette value of 1. This cluster examines the role of transport phenomena, such as the movement of fluids and particles, in tumor growth and the potential for magnetotherapy to influence these processes. Contributors like Rashevsky and Garcia-Sainz have explored the mathematical and physical aspects of magnetotherapy in oncology. Cluster #19, with 15 members and a silhouette value of 0.999, is labeled Therapeutic Modalities. This cluster explores various therapeutic modalities in hand surgery, including the use of electromagnetic fields. Contributors like Basford JR and Bommannan D have played significant roles in advancing the application of electromagnetic fields in surgical and rehabilitative contexts.

The 20th largest cluster, Spontaneous Abortion, has 14 members and a silhouette value of 1. This cluster examines the relationship between magnetic fields and reproductive health, particularly in the context of spontaneous abortion. Authors like Cremer-Bartels G and Czeisler CA have contributed to understanding how magnetic fields might influence reproductive outcomes. Cluster #21, labeled Breast Cancer, consists of 13 members and has a silhouette value of 0.994. This cluster explores the use of magnetotherapy in the rehabilitation of breast cancer patients. Contributors like Ponomarenko GN and Gerasimenko MYU have explored the potential benefits of electromagnetic fields in cancer recovery and rehabilitation. The 22nd cluster, Fat Disruption, has six members and a silhouette value of 0.999. This cluster focuses on using electromagnetic fields to disrupt fat tissues, a technique used in body contouring and weight management. Contributors like Kinney BM and Duncan D have advanced the understanding of how electromagnetic fields can be applied in aesthetic medicine.

Most Relevant Sources

Table 3 highlights the most relevant sources in magnetotherapy research, with Problems of Balneology, Physiotherapy and Therapeutic Physical Culture leading the list with 116 articles, emphasizing its focus on therapeutic practices. Electromagnetic biology and medicine (57 articles) and bioelectromagnetics (55 articles) are crucial for understanding the biological effects of electromagnetic fields. Other sources like the Bulletin of Rehabilitation Medicine (25 articles) and Przeglad Elektrotechniczny (24 articles) indicate the application of magnetotherapy in rehabilitation and its intersection with engineering disciplines. The multidisciplinary reach of magnetotherapy is reflected in journals like Plos One (21 articles) and Scientific Reports (19 articles). Additionally, the presence of Biomedical Engineering (19 articles) and the International Journal of Radiation Biology (19 articles) underscores its relevance in medical technology and biological studies. Including 18 articles in the Cochrane Database of Systematic Reviews indicates magnetotherapy's rigorous evaluation and clinical significance, highlighting its wide-ranging applications across clinical and technical domains.

**Table 3:** Most relevant sources The table lists the most relevant sources in the field, highlighting the key journals and sources contributing to the research area.

Sources	Article
Problems of Balneology, Physiotherapy and Therapeutic Physical Culture	116
Electromagnetic Biology and Medicine	57
Bioelectromagnetics	55
Bulletin of Rehabilitation *Medicine*	25
Przeglad Elektrotechniczny	24
Plos One	21
Biomedical Engineering	19
International Journal of Radiation Biology	19
Scientific Reports	19
Cochrane Database of Systematic Reviews	18

Countries' Scientific Productions

**Figure 4:**
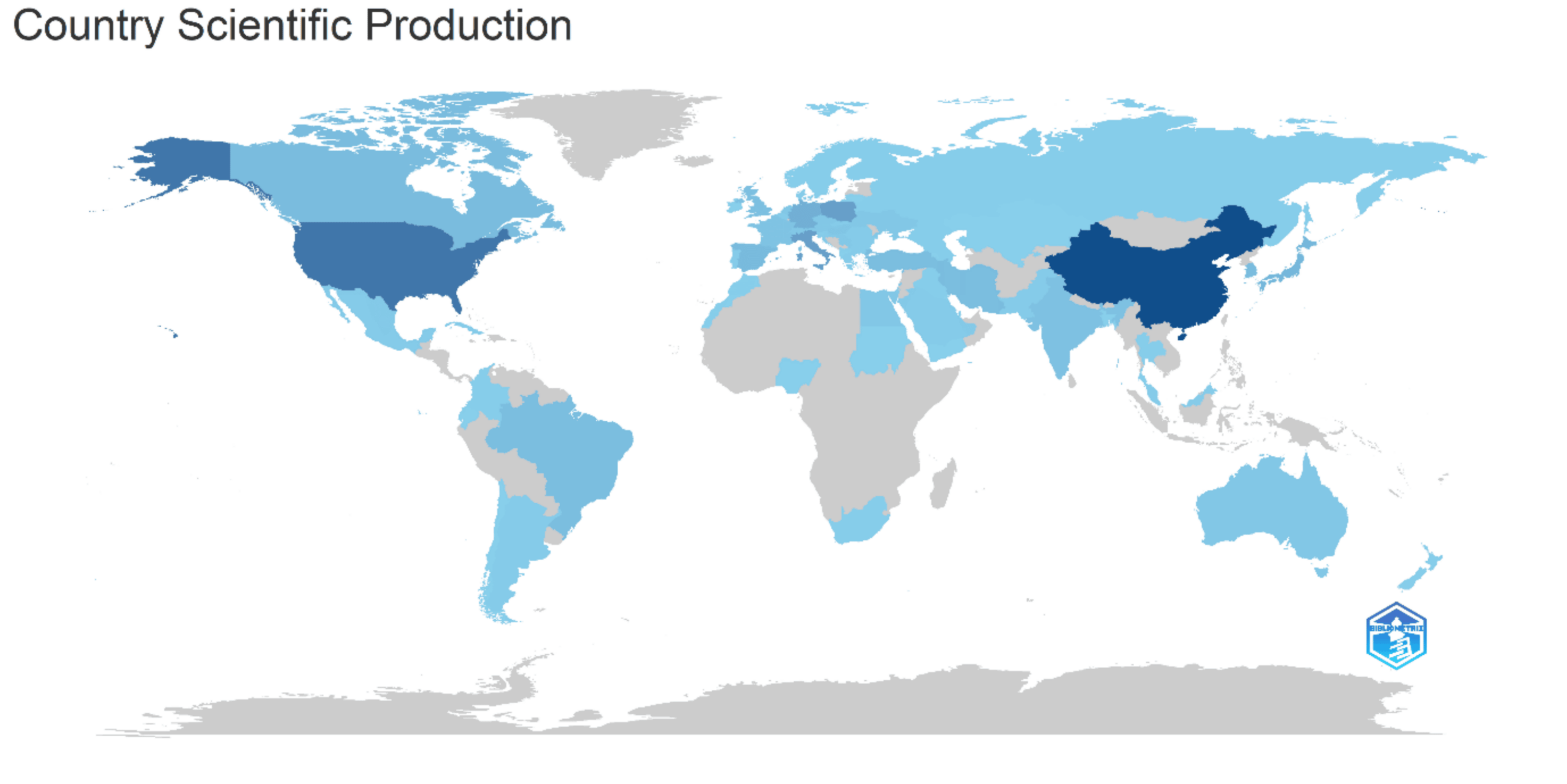
Countries' scientific productions The figure presents a world map illustrating the scientific production by country, where darker shades of blue indicate higher levels of research output, highlighting key contributors such as China, the United States, and Russia.

Figure 4 illustrates the global distribution of scientific production, highlighting the contributions of different countries. Darker shades of blue indicate higher levels of scientific output, while lighter shades represent lower levels. China leads with 1999 articles, indicating its dominant role in this field. The USA follows with 1335 articles showcasing significant research activity. Italy and Poland also make notable contributions, with 701 and 678 articles, respectively. Germany (444 articles) and Japan (330 articles) are key contributors in Asia and Europe. Other countries with substantial outputs include Spain (271 articles), South Korea (265 articles), Canada (258 articles), and Iran (245 articles).

Timeline Network Visualization of Countries' Collaborations

**Figure 5:**
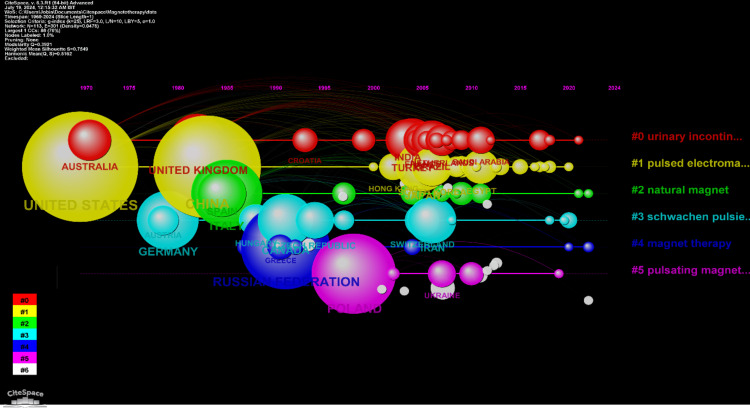
Timeline network visualization of countries' collaborations The figure shows a timeline network visualization of international collaborations, where the size of the nodes represents the number of publications, the color represents different clusters of research topics, and the lines indicate collaborative links between countries over time, with prominent collaborations involving countries like Australia, the United Kingdom, and the United States.

Figure 5 presents the timeline network visualization of countries' collaborations in magnetotherapy research which reveals seven distinct clusters, each reflecting unique collaborative efforts and research focuses. The largest cluster (#0), labeled as "Urinary Incontinence," comprises 20 members with a silhouette value of 0.724. This cluster's major citing article by Zhang (2010) highlights the management of hip and knee osteoarthritis and involves significant contributions from the United Kingdom, Turkey, and India [31]. Cluster #1, "Pulsed Electromagnetic Field," also has 20 members but with a silhouette value of 0.656. This cluster features a major citing article by Shen (2017) on nanoparticle aggregates in cancer cells, with prominent collaborations from the United States, China, and Japan [32]. Cluster #2, "Natural Magnet," includes 14 members and boasts a silhouette value of 0.864, focusing on low-frequency magnetic fields and chronic neck conditions, with major contributions from Italy, Spain, and Serbia. The fourth cluster (#3), labeled "Schwachen Pulsierenden Magnetfeldern," has 12 members with a silhouette value of 0.856, centering on magnetic fields in cancer diagnostics and involving Germany, Canada, and Iran. Cluster #4, "Magnet Therapy," consists of eight members with a silhouette value of 0.552, examining the efficiency and safety of magnet therapy for osteoarthritis, with significant input from the Russian Federation, Greece, and the USA. The sixth cluster (#5), "Pulsating Magnetic Field," also with eight members and a silhouette value of 0.84, focuses on hip osteoarthritis and includes contributions from Poland, Ukraine, and Romania. Finally, cluster #6, "Magnetic Liposome," the smallest with four members and a silhouette value of 0.961, delves into the effectiveness of physical and electrophysiotherapy for lateral epicondylitis, with notable contributions from Taiwan, the University of Michigan Medical School, and Chang Gung Memorial Hospital and Chang Gung University.

Historiograph

**Figure 6:**
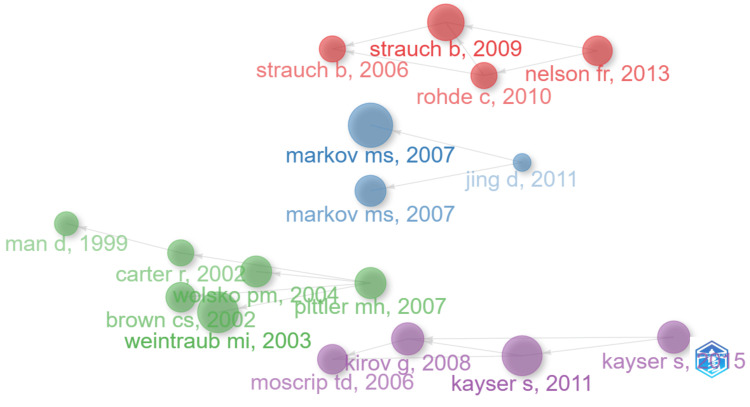
Evolution and development of publications The figure presents a historiograph illustrating the evolution and development of key publications over time. The nodes represent influential papers, with their size indicating the number of citations. The connections between nodes show citation relationships, tracing the development of research ideas and contributions from early foundational works to more recent studies, highlighting major contributors.

Figure 6 shows a historiograph that visualizes the citation relationships and the evolution of key publications in magnetotherapy research over time. The nodes represent significant publications, with their size indicating the number of citations received. The arrows denote citation links between publications, illustrating how research builds upon previous studies. The historiograph is divided into several clusters, each represented by different colors (green, blue, red, and purple), indicating distinct research themes or areas within magnetotherapy. The green cluster includes early foundational works such as "Man D, 1999," connecting to subsequent important studies like "Carter R, 2002," "Wolsko PM, 2004," and "Pittler MH, 2007," forming a core body of early research. The blue cluster prominently features publications by "Markov MS, 2007," showing significant influence on later research, including "Jing D, 2011." The repeated appearance of "Markov MS, 2007" suggests its widespread impact on various aspects of magnetotherapy. The red cluster highlights interconnected works by "Strauch B, 2006" and "Strauch B, 2009," leading to influential studies like "Rohde C 2010" and "Nelson FR 2013," indicating a focused research area that evolved over time. The purple cluster centres around publications by "Kayser S, 2011" and "Kayser S, 2015," showing their pivotal role in this research trajectory, with contributions from significant works like "Kirov G, 2008" and "Moscrip TD, 2006," reflecting ongoing interest and development in specific subfields of magnetotherapy.

Trend Topics

**Figure 7:**
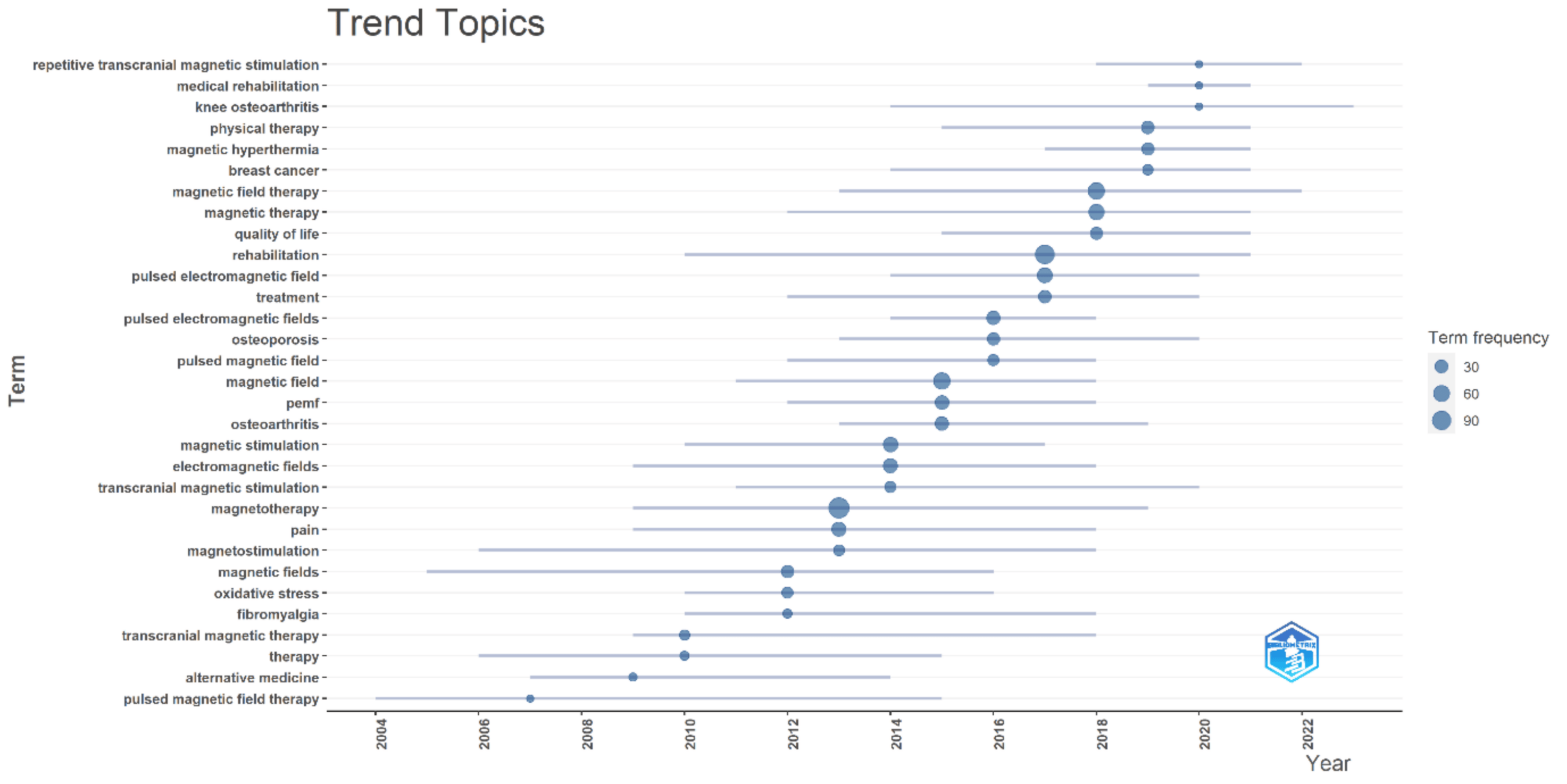
Visualization of trending topics The figure visualizes trending topics in the research field over time, where each line represents a term's presence across years, and the size of the dots indicates the frequency of that term in the literature, highlighting the rise and fall of various research interests with the most recent trends shown towards the right side of the graph.

Figure 7 illustrates the trending topics over 20 years from 2004 to 2024, with a word minimum frequency of 10. Each term's frequency is represented by the size of the corresponding bubble, with larger bubbles indicating higher frequencies. The horizontal axis represents the timeline, and the vertical axis lists the research terms, demonstrating their emergence and prominence over the years [33]. The figure highlights the evolving focus in magnetotherapy research. Early foundational terms like "magnetotherapy," "magnetic fields," and "pain" were prevalent around 2004-2006. As the field developed, more specialized terms such as "pulsed magnetic field therapy," "transcranial magnetic stimulation," and "fibromyalgia" gained prominence around 2010-2012, reflecting a shift towards specific applications and therapies. In recent years, from 2015 onwards, there has been a notable increase in the diversity of research topics, with terms like "repetitive transcranial magnetic stimulation," "medical rehabilitation," "knee osteoarthritis," and "quality of life" becoming prominent. This indicates a growing interest in the therapeutic applications of magnetotherapy for various health conditions and improvements in quality of life. The frequency and timing of these terms illustrate ongoing advancements and an expanding scope in magnetotherapy research.

Thematic Map

**Figure 8:**
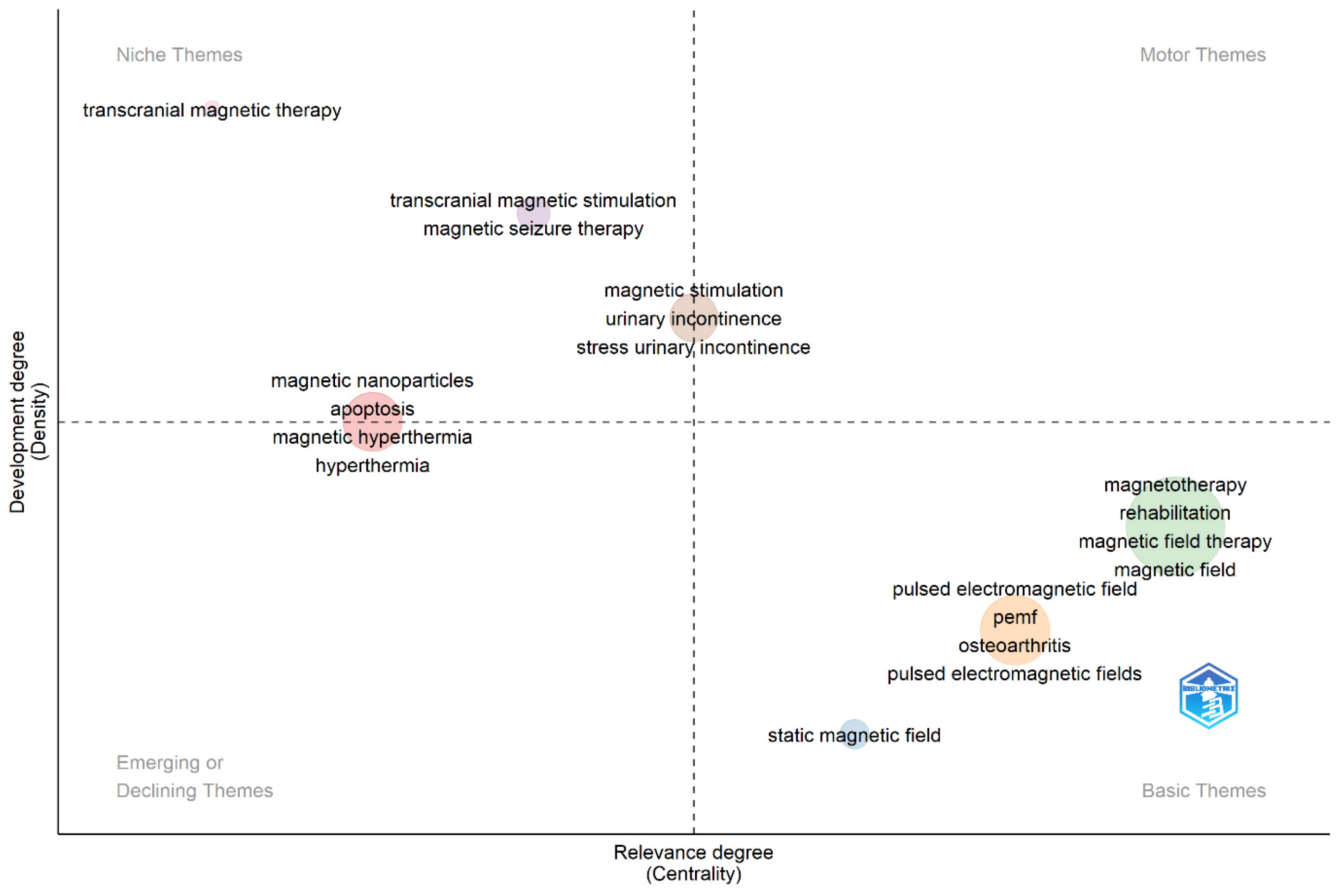
Thematic visualization of keywords The figure provides a thematic visualization of keywords, mapping them across four quadrants based on their relevance (centrality) and developmental stage (density).

Figure 8 provides a thematic map that visualizes the development and relevance of keywords in magnetotherapy research. The map is divided into four quadrants based on two dimensions: the degree of development (density) on the vertical axis and the degree of relevance (centrality) on the horizontal axis [34]. This classification helps in understanding the importance and maturity of different research themes. In the Motor Themes quadrant (upper right), keywords such as magnetotherapy, rehabilitation, and magnetic field therapy are found. These themes are well-developed and highly relevant, indicating significant research activity and practical applications. These central topics drive the field of magnetotherapy and reflect its core focus areas. The Niche Themes quadrant (upper left) includes terms like transcranial magnetic stimulation, magnetic seizure therapy, depression, magnetic nanoparticles, apoptosis, and magnetic hyperthermia. These themes are highly developed but less central, indicating specialized areas of research that, while significant, may not be widely connected to other topics in the field. These advanced, focused research areas have specific applications and are critical within their niche. In the Basic Themes quadrant (lower right), keywords such as pulsed electromagnetic field, osteoarthritis, static magnetic field, and pemf (pulsed electromagnetic fields) are present. These themes are highly relevant but less developed, forming the foundation of magnetotherapy research. They represent broad topics underpinning many studies, with potential for further exploration and development. Lastly, the Emerging or Declining Themes quadrant (lower left) contains terms such as transcranial magnetic therapy. These themes are less developed and less relevant, suggesting they are either emerging areas of research that are yet to gain traction or declining topics that are losing interest within the research community. Overall, the thematic map highlights the diversity and evolution of research in magnetotherapy, indicating both established and emerging areas of interest.

Keywords With Strongest Citation Burst

**Figure 9:**
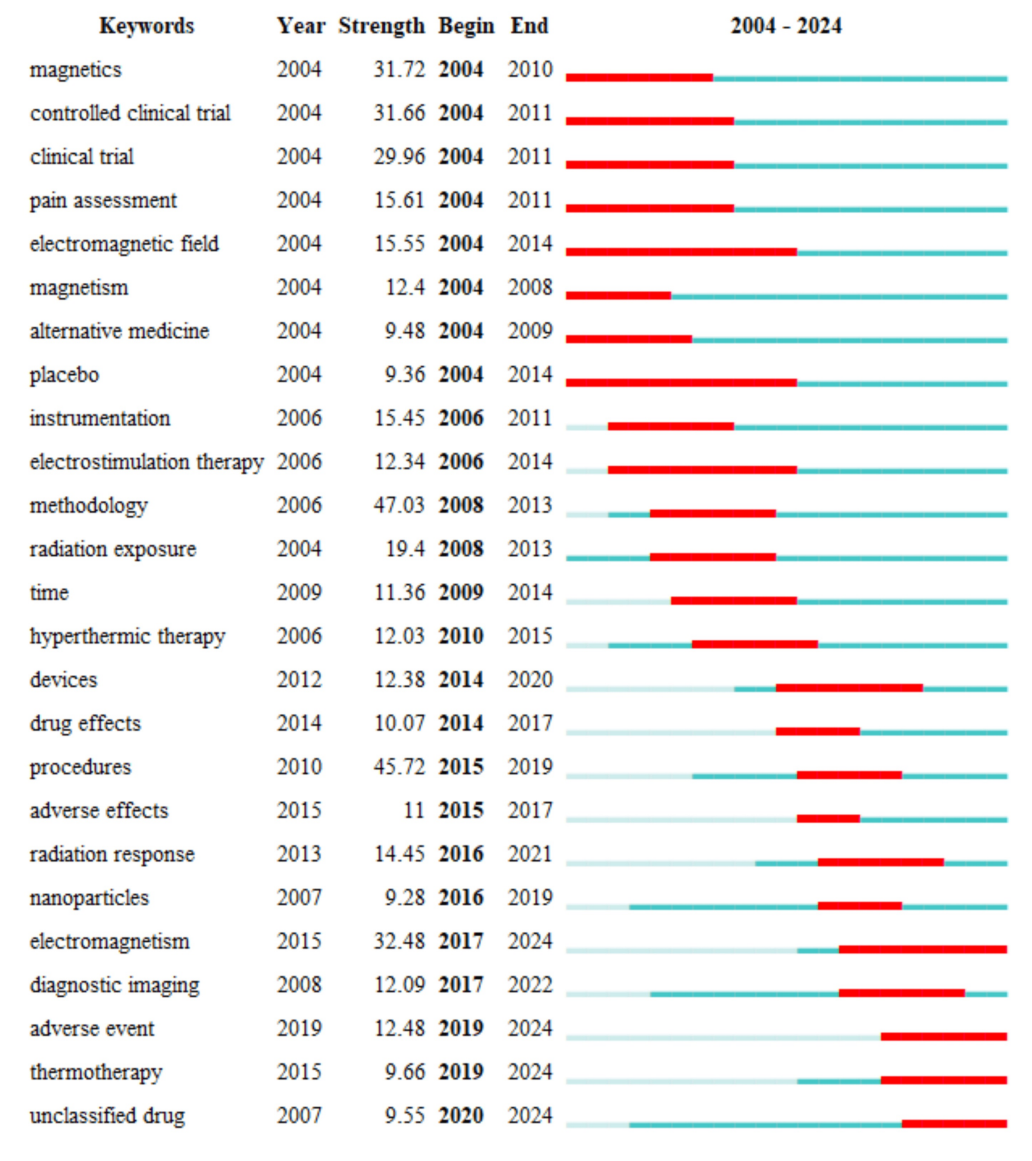
Top 25 keywords with strongest citation bursts The figure displays the top 25 keywords with the strongest citation bursts, indicating periods when these keywords experienced a significant increase in attention. The red bars represent the duration of the burst, showing when each keyword gained prominence, while the blue segments indicate periods of lower citation activity.

Figure 9 highlights the top 25 keywords in magnetotherapy research that exhibited the strongest citation bursts between 2004 and 2024. These citation bursts represent periods when particular keywords received intensified attention within the academic community, reflecting significant trends or shifts in research focus over time [35]. Early research (2004-2014) concentrated on foundational aspects of magnetotherapy, as indicated by keywords like "magnetics," "controlled clinical trial," "clinical trial," and "electromagnetic field." During this period, there was also notable interest in assessing the effectiveness of magnetotherapy in pain management, as evidenced by the citation bursts for "pain assessment" and "placebo." As the field progressed between 2006 and 2014, there was a noticeable shift toward improving the methodologies and instrumentation used in magnetotherapy research. Keywords such as "instrumentation" and "methodology" saw strong citation bursts during this period, indicating a focus on refining the tools and approaches that underpin magnetotherapy studies. Additionally, the emergence of "electrostimulation therapy" during this phase highlights the growing exploration of magnetotherapy's applications in related therapeutic areas. Further, attention to "radiation exposure" and "time" suggests growing concerns about the safety and duration of magnetotherapy treatments, especially in relation to the use of magnetic fields. From 2014 onwards, there was a distinct shift toward more specialized clinical applications and safety considerations, with keywords like "devices," "drug effects," "adverse effects," and "procedures" gaining prominence. This period also saw the integration of magnetotherapy with other treatments, as indicated by the emergence of "hyperthermic therapy" and "thermotherapy." In recent years (2017-2024), newer research areas such as "nanoparticles" and "diagnostic imaging" have come to the forefront, reflecting the intersection of magnetotherapy with advanced technologies like nanotechnology and medical imaging. Ongoing research interest in keywords like "adverse event," "unclassified drug," and "thermotherapy" suggests a continued focus on the safety, efficacy, and novel applications of magnetotherapy, highlighting the field's evolution and increasing sophistication.

Discussion and research gaps

The focus on specific applications of magnetotherapy has been evolving, but there are few long-term studies examining the sustained effects and efficacy of various magnetotherapy treatments. Future research should prioritize conducting longitudinal studies to establish solid proof of the long-term benefits and possible side effects of these therapies. While certain areas, such as magnetic nanoparticles and magnetic hyperthermia, are well developed in specialized areas, they are not sufficiently correlated with broader magnetotherapy research. This points to a gap in interdisciplinary studies, which could align these specialized fields with the wider applications of magnetotherapy. Through this integration, new therapeutic strategies could be developed, and a more comprehensive understanding of the field could be achieved. Divergent terms and emerging themes indicate a lack of standardization of treatment protocols in magnetotherapy. Research often adopts different methodologies without a unified framework, leading to fragmented findings. In the future, studies should focus on developing and validating standardized protocols to ensure the replicability and reliability of results across different settings and populations. While advanced research areas have examined the mechanisms of apoptosis and magnetic hyperthermia, the cellular and molecular mechanisms involved in the modes of action in different magnetotherapies are not yet fully understood. Further studies into these mechanisms are necessary to optimize treatment effectiveness and tailor therapy according to the individual patient's needs. In recent years, the integration of the Internet of Things (IoT) into healthcare has significantly advanced medical practices, including magnetotherapy. IoT enables real-time monitoring and personalized treatment adjustments, enhancing the precision and efficacy of magnetotherapy sessions. Wearable devices equipped with sensors can track patient responses to magnetic fields, allowing for dynamic adjustments to treatment protocols based on individual feedback. As seen in the Internet of Surgical Things (IoST), this real-time data flow can optimize outcomes and reduce errors in treatment delivery [36].

Practical implications

The increasing use of terms such as "pulsed electromagnetic field therapy," "transcranial magnetic stimulation," and "medical rehabilitation" demonstrates a significant amount of research being conducted in the field of magnetotherapy clinical applications. The progress made in this area can lead to improved clinical practices, which can enhance patient outcomes in terms of pain management, rehabilitation, and treatment for chronic conditions such as osteoarthritis and fibromyalgia. As concerns about quality of life and rehabilitation continue to grow, magnetotherapy could become a standard part of healthcare policy and practice. It would be beneficial for policymakers and healthcare professionals to consider integrating magnetotherapy into treatment programs, particularly for conditions where other forms of therapy may be insufficient.

The focus of research on emerging technologies, such as magnetic nanoparticles and advanced magnetotherapeutic devices, suggests huge potential for innovation in medical devices. These collaborations can lead to the development of next generation magnetotherapy devices with more effective personalized treatment options. With the increasing scope and applications of magnetotherapy, there is a need for specialized educational and training programs for health professionals. Research findings on magnetotherapy should be updated, and practical training should be provided on the subject at medical institutions and further education establishments. This would enable health professionals to apply these therapies appropriately. Addressing these research deficiencies and implementing these practical implications will likely propel the field of magnetotherapy forward for a wide variety of health conditions if used appropriately.

## Conclusions

This bibliometric study has been conducted in the field of magnetotherapy to map the research landscape and focus on major trends, influential authors, and collaborative networks. This analysis realizes areas of huge research activities with significant gaps that should be addressed to advance this field. One major finding is a lack of long-term studies with standardized treatment protocols, which might hamper the consistency and reliability of the research outcomes. In particular, longitudinal studies should be the mainstay of future research in order to firmly chart out the persistence of magnetotherapy treatments and their efficiency. Moreover, the development and validation of standardized protocols will ensure replicability across different settings and populations. This also requires interdisciplinary studies wherein specialized areas are integrated, such as magnetic nanoparticles and magnetic hyperthermia, with larger applications of magnetotherapy, to come up with newer therapeutic strategies that are innovative in nature. Finally, a policy on magnetotherapy in mainstream healthcare and specialized educational programs for health professionals can improve the clinical applications of these therapies. Taking on board the recommendations provided herein to address these gaps can realize the full potential that magnetotherapy has to offer for patients' benefit and the progress of medical science.
